# Seasonality in associations between dietary diversity scores and nutrient adequacy ratios among pregnant women in rural Malawi – a cross-sectional study

**DOI:** 10.29219/fnr.v63.2712

**Published:** 2019-02-27

**Authors:** Katrine G. Hjertholm, Gerd Holmboe-Ottesen, Per O. Iversen, Ibrahimu Mdala, Alister Munthali, Kenneth Maleta, Zumin Shi, Elaine Ferguson, Penjani Kamudoni

**Affiliations:** 1 Department of Nutrition, Institute of Basic Medical Sciences, University of Oslo, Oslo, Norway; 2 Department of Community Medicine and Global Health, Institute of Health and Society, University of Oslo, Oslo, Norway; 3 Division of Human Nutrition, Stellenbosch University, Tygerberg, South Africa; 4 School of Public Health and Medicine, University of Malawi, Zomba, Malawi; 5 Discipline of Medicine, School of Medicine, Faculty of Health Sciences, University of Adelaide, Adelaide, Australia; 6 Department of Population Health, London School of Hygiene and Tropical Medicine, London, United Kingdom

**Keywords:** dietary diversity score, nutrient adequacy ratio, pregnancy, seasonality, sub-Saharan Africa

## Abstract

**Background:**

Dietary diversity scores (DDS) are simple indicators often used as proxies for nutrient adequacy. A 10-food group indicator is proposed by the Food and Agriculture Organization of the United Nations as a global standard for measuring dietary diversity among women in low-resource settings. However, its validity as a proxy for nutrient adequacy across different agricultural seasons for pregnant women has not been determined.

**Objective:**

We studied associations between DDS and nutrient adequacy ratios (NAR) across two different agricultural seasons (pre- and post-harvest seasons) for pregnant women in rural Malawi and assessed whether a 1-day DDS or a 3-day DDS would be the best indicator of nutrient adequacy.

**Design:**

Dietary intakes of 330 pregnant women were assessed between gestational weeks 28 and 35. Intakes of energy, macronutrients, and 11 micronutrients were estimated using three repeated interactive 24-h diet recalls, and DDS were also calculated from these days. Correlation coefficients (*r*) between DDS, NAR, and mean adequacy ratio (MAR) of the 11 micronutrients were determined.

**Results:**

After energy adjustments, we found significant correlations between DDS and MAR with both DDS indicators in the preharvest season (*r* = 0.22–0.23; *p* < 0.001) but not in the post-harvest season (*p* > 0.05). For individual energy-adjusted NARs, correlations were not consistently significant across the two seasons and the two DDS indicators.

**Conclusions:**

Our results suggest that DDS could be used to predict overall nutrient adequacy during the preharvest season. As similar correlations were found using both the 1- and 3-day indicators, we recommend using a 1-day DDS, for simplicity. However, as the indicators are sensitive to seasonality they should be used with care in this study setting.

## Popular scientific summary

- In this study we found that dietary diversity and nutrient intakes vary between agricultural seasons among pregnant women in rural Malawi.
- Dietary diversity is often used as a proxy for nutrient adequacy. However, our results show that the relationship between dietary diversity and nutrient adequacy is affected by seasonality.
- We recommend that care is taken when using dietary diversity as a proxy for nutrient adequacy across seasons in this setting.

## Introduction

Undernutrition poses a serious intergenerational challenge in sub-Saharan Africa (1). Poor maternal nutritional status is an important determinant of intrauterine growth restriction and small neonatal size, implying increased risk of mortality and morbidity for both mothers and their newborns (2). Previous studies have uniformly shown that Malawian pregnant women have inadequate intakes of most nutrients (3–5). Dietary intakes of micronutrient-rich foods such as meat, eggs, and milk are low, and approximately three-quarters of the food energy comes from maize, the main staple food, usually served as a thick porridge *nsima*. During pregnancy, it is particularly important to consume nutrient-dense foods because requirements for most nutrients are higher than in the non-pregnant state (6).

Information on dietary intakes is needed for designing, targeting, and evaluating programs with the aim of improving nutritional status (7). Collection of quantitative data on dietary intakes of energy and nutrients is complex and costly (7). Consequently, very few developing countries conduct nationally representative dietary surveys providing such information (8). It has been shown that increasing food group diversity in the diet is positively associated with nutrient intakes and thus will likely promote good health (9). Arimond et al. found that a dietary diversity score (DDS), which is a simple count of food groups consumed over a defined time period, is associated with nutrient adequacy (achievement of recommended intakes of energy and essential nutrients) among poor women of reproductive age (including pregnant and lactating) in five countries (8). This work resulted in a recommendation for the dichotomous minimum dietary diversity for women (MDD-W) indicator, where a DDS of ≥5 out of 10 food groups is set as a cutoff point for acceptable nutrient adequacy (10).

Farming communities in Malawi, as in low income countries in general, experience seasonal changes in food availability and access, which often coincide with variations in household income and expenditures. These yearly cycles affect the adequacy of nutrient intakes for everyone but have special repercussions for pregnant women, with their increased nutritional needs (3). Using DDS as a proxy for nutrient adequacy thus necessitates consideration for seasonality. To the best of our knowledge, it has not been adequately addressed whether the proportion of pregnant women achieving MDD-W varies across seasons and whether seasonality affects the association between DDS and nutrient adequacy in a resource-poor setting.

A reference period of 24 h is most frequently used when measuring DDS. This does not necessarily provide information about an individual’s habitual diet (10). We therefore compared a 1-day DDS and 3-day DDS to determine if the latter would be a better indicator of nutrient adequacy across seasons among pregnant women in the rural Malawian setting, as it is expected to be more closely associated with habitual nutrient intakes.

## Materials and methods

### Study area and participants

A cross-sectional study was conducted in the rural Nankumba Traditional Authority of Mangochi District. This area has a population of about 150,000 people; and the most common occupations are subsistence farming and fishing. Data were collected from pregnant females during the two agricultural seasons: the post-harvest season (August through September 2013), when food availability is sufficient for most households, and the preharvest season (February through March 2014), when many households experience food shortage.

### Sample and study design

This study was part of a larger research project where one of the outcomes was iron deficiency. The sample size was thus calculated to detect the prevalence of iron deficiency within ±10%, at a 95% confidence level, assuming a design effect of 1.5 and a 35% rate of attrition. A design effect of 1.5 was added to account for similarities between participants from the same geographic clusters (villages) (11). Pregnant females were recruited to the study using a one-stage randomized sampling procedure (12). Initially, 76 clusters, defined by geographic areas, were randomly selected. From each selected cluster, four to six eligible participants were identified by health volunteers or health surveillance assistants. Only females between 28 and 35 weeks of gestation were recruited because the delivery had to take place within the current agricultural season, and 10 days were required for data collection from each individual. Gestational age was estimated from the reported last menstrual period or through fundal height. Females with severe illness or those who had not been a resident in the Nankumba area for the previous 6 months were excluded. Participants were recruited from two separate recruitment rounds: post-harvest season (August through September 2013) and preharvest season (February through March 2014).

### Collection of dietary data

**Fig. 1:**
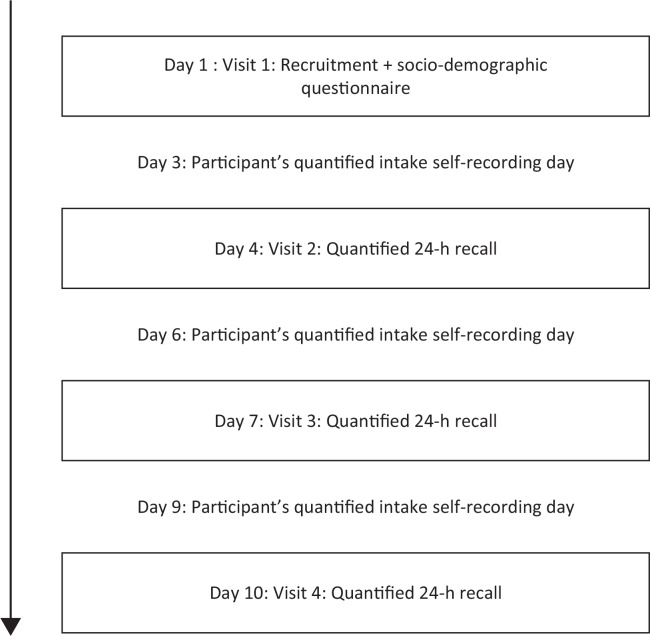
Overview of the interview process. The boxes represent interviews performed during home visits. The intake recording days between the interviews are the days that the participants were interviewed about. In total there were 3 days with information on quantified dietary intake. Intakes on these days were used to calculate mean nutrient intake used for mean adequacy ratio and nutrient adequacy ratios. The 1-day DDS was constructed from intakes from the first day of ‘participant’s quantified intake self-recording day’; while the 3-day DDS was constructed from intakes from all 3 days.

During a 10-day period we collected quantitative dietary recall data from the participants (Fig. 1). The interviews took place in their homes in order to encourage participation and improve recall of the foods consumed (13). The quantified recall data were collected by three repeated interactive multipass 24-h recalls (13), where food and beverage portion sizes were estimated by weighing food models (consisting of real food) on a digital kitchen scale (precision ± 3 g) and recorded. To improve visualization of portion sizes, participants were provided with bowls and cups and asked to use them instead of eating from shared plates with other household members. For composite dishes we collected detailed information on the weight of each ingredient. In cases where the participants did not know the amount of ingredients, we used average recipes typical for the area. To enhance memory for the recalls, participants were asked to mark on pictorial charts all foods and beverages consumed prior to the day of interview (Supplementary Fig. 1).

During the first visit (Day 1), the participants were given bowls, cups, and pictorial charts to record their food consumption over the subsequent days, and they were trained how to use them. The participants were then revisited on Days 4, 7, and 10 out of the 10-day period. During these interviews, the quantified interactive multiple pass 24-h recall data were collected, and food portion sizes were estimated. The pictorial charts were then collected, and any discrepancies between the recalled and recorded dietary intakes were resolved. On Days 4 and 7, other sets of blank pictorial charts were given to the participants to record (with check marks) their dietary intakes before the next visit.

### Collection of sociodemographic data

A precoded questionnaire developed and used in rural Malawi previously was administered at the first visit to collect sociodemographic variables on both participant and household level, such as age, education level, occupation, number of children, number of people in the household, and food security. As a proxy for household economic status, a household asset index was calculated based on 11 household items given scores according to their monetary value (14).

### Dietary data evaluation

The quantified food intake data were converted to energy and nutrient intakes using food composition tables (FCT). As there is no official FCT for Malawi, the FCT used in this study was an unpublished construct made from a combination of several FCTs, including the US Department of Agriculture’s food composition database, the West African FCT, and the Lesotho FCT (15–17). Average intakes of the 3 days were calculated for energy, macronutrients, and 11 micronutrients (vitamin A, vitamin C, thiamin, riboflavin, niacin, folate, vitamin B_12_, vitamin B_6_, calcium, iron, and zinc). Usual intakes were estimated using the software program PC-SIDE, and the percentage of women at risk of inadequate nutrient intakes were evaluated using the estimated average requirement cut-point method, which is recommended for evaluating group intakes (18).

DDS is defined as the number of food groups consumed over a reference period and used as a proxy for population-level dietary adequacy or for monitoring changes over time (19). To estimate the DDS, food items were assigned to the 10 food groups used for the MDD-W, which were 1) grains, white roots, and tubers; 2) pulses; 3) nuts and seeds; 4) dairy; 5) meat, poultry, and fish; 6) eggs; 7) dark green leafy vegetables; 8) vitamin A-rich fruits and vegetables; 9) other vegetables; 10) other fruits. No minimal amount was required for a food item to be included. Each food group was weighted equally with the value 1. MDD-W was achieved with a score of 5 or more. We used two different reference periods for DDS to investigate correlation with nutrient adequacy: a 3-day DDS compiled from 3 days of dietary data, and a 1-day DDS, where the first day of dietary data was chosen.

### Correlation between DDS and nutrient adequacy

To investigate correlation coefficients (*r*) between dietary diversity and nutrient adequacy, nutrient adequacy ratios (NARs) were calculated for the 11 micronutrients by dividing the participants’ actual intakes of each nutrient (3-day mean intakes) by the recommended daily allowance (RDA) for that nutrient. We used the Institute of Medicine’s RDAs for pregnant females age category 19–30 years (18). Low bioavailability was assumed for iron and zinc. A NAR equal to 0 indicates a diet devoid of that nutrient, whereas a NAR equal to 1 indicates a diet that achieved or exceeded the recommended nutrient intake for that nutrient. To obtain an overall estimate of the nutritional adequacy a mean adequacy ratio (MAR) was calculated from the 11 NARs according to the following formula:

$$\text{MAR:}\frac{\text{∑ NAR}\left(\text{each truncated at 1}\right)}{\text{Number of nutrients}}$$

Each NAR was truncated at 1 to avoid the possibility of a nutrient with a high NAR to compensate for a nutrient with a low NAR. The maximum possible MAR value was therefore 1 and the minimum was 0.

### Statistical methods

Categorical variables are presented as proportions, and continuous variables are presented as means and standard deviations (SD) if normally distributed or median and interquartile range if not normally distributed. Participants’ data were nested within geographical units. Estimates of intracluster correlation obtained from both the variance components models and full models showed absence of variability between these geographical units. Pearson’s chi-square tests were applied to compare MDD-W between seasons, whereas *t*-tests were used to compare mean DDS between seasons. To investigate the associations between DDS and NARs, correlation analyses were conducted. All data were log transformed to meet the criteria for Pearson’s correlations. Partial correlations were conducted to adjust for energy intake. Statistical analyses were conducted using SPSS Statistics for Windows, version 22.0, and Stata/SE 14.0. A *p*-value ≤ 0.05 was considered statistically significant.

### Study approvals

This project was approved by the College of Medicine Research and Ethics Committee in Malawi and the Regional Committee for Medical and Health Research Ethics in Norway. In order to sensitize the local community to the study, chiefs from all villages of the Nankumba traditional authority were invited to an information meeting held by the study coordinator. The participants signed a consent form, either by signature or by fingerprint. Soap was given as an incentive after each interview and the participants also received a bag of sugar after the last interview.

## Results

### Participant characteristics

In the post-harvest season 229 females were invited to participate. Of these, 223 accepted and 203 completed all dietary interviews. In the preharvest season 133 were invited to participate; of these, 130 accepted, and 127 completed all interviews. The mean age of the participants was 25 years in both seasons (range 13–43 years in the post-harvest season and 15–43 years in the preharvest season). Most participants were married (86% in the post-harvest season and 85% in the preharvest season) and living in households where the husband was the household head. Most participants (72% in the post-harvest season and 80% in the preharvest season) were literate. Subsistence farming was the most common occupation for the participants and their husbands. There were no significant differences between the two groups in these characteristics.

### Seasonality in dietary intakes

A high percentage of participants were at risk of inadequate intakes of several nutrients across both seasons (Table 1). For zinc, riboflavin, and thiamin, the percentages of participants at risk of inadequate intakes were higher in the preharvest season. The mean (SD) 3-day DDS was 6.5 (1.2) in the post-harvest season and 5.8 (1.4) in the preharvest season. The mean (SD) 1-day DDS was 4.7 (1.2) in the post-harvest season and 4.0 (1.0) in the preharvest season. In the post-harvest season, 57.6% achieved MDD-W, whereas in the preharvest season, 33.3% achieved it. The differences between the seasons were statistically significant (*p* < 0.001 in all cases).

**Table 1:** Percentage of participants at risk of inadequate nutrient intakes (*n* = 330)

Nutrient	Estimated average requirements (Institute of Medicine)	Preharvest season (*n* = 127)	Post-harvest season (*n* = 203)	*p*
Protein	50 g	40.8	40.5	0.9
Calcium	800 mg	100	100	–
Iron^a^	5% absorption	95.8	94.6	0.5
Zinc^b^	12 mg	96.2	90.2	0.05
Vitamin C	70 mg	18.5	23.6	0.3
Thiamin	1.2 mg	41.4	20.7	<0.001
Riboflavin	1.2 mg	99.9	93.7	0.004
Niacin	14 mg	77.2	79.7	0.6
Folate	520 μg DFE (dietary folate equivalent)	100	99.9	–
Vitamin A	550 RAE (retinol activity equivalent)	96.9	95.3	0.4
Vitamin B12	2.2 μg	Not available^c^	Not available	Not available
Vitamin B6	1.6 mg	54.4	47.2	0.2

In cases where all participants are at risk of inadequate intakes, no *p*-values are given.

a Analyzed using the full probability approach, assuming 5% absorption for iron.

b Assumed low bioavailability for zinc.

c Because of 0 values the data could not be transformed to normality and estimation of percentage at risk of inadequate intake could not be calculated.

### Correlation between DDS and NARs

When using the 3-day DDS we found a positive association with NAR (adjusted for energy) for calcium, vitamin C, vitamin A, vitamin B_6_, and MAR in the preharvest season, whereas in the post-harvest season DDS was associated with vitamin C, riboflavin, folate, and vitamin A (Table 2). Vitamin B_12_ was negatively correlated with a 3-day DDS in the post-harvest season. The 1-day DDS was positively correlated with MAR and NAR for calcium, vitamin C, vitamin A, and vitamin B_6_ in the preharvest season (Table 3). In the post-harvest season, the 1-day DDS was not correlated with MAR. The only correlation with individual NARs was the negative correlation with NAR for vitamin B_12_.

**Table 2:** Correlations between the 3-day dietary diversity score and nutrient adequacy ratios among the participants (*n* = 330)

	Pre-harvest season (*n* = 127)		Post-harvest season (*n* = 203)	
Nutrient adequacy ratio	Adjusted Pearson’s correlation coefficient^a^	*p*	Adjusted Pearson’s correlation coefficient^a^	*p*
Calcium	0.29	<0.01	0.56	0.59
Iron	0.07	0.35	0.08	0.43
Zinc	0.06	0.40	0.09	0.34
Vitamin C	0.40	<0.01	0.25	0.01
Thiamin	0.04	0.59	0.15	0.12
Riboflavin	0.10	0.21	0.19	0.05
Niacin	0.17	0.02	−0.03	0.75
Folate	0.33	<0.01	0.47	<0.01
Vitamin A	0.48	<0.01	0.29	<0.01
Vitamin B_12_	−0.14	0.06	−0.28	<0.01
Vitamin B_6_	0.16	0.04	0.08	0.38
Mean adequacy ratio	0.23	<0.01	0.13	0.20

a All data were log-transformed to meet the criteria for Pearson’s correlation. Partial correlations were conducted to adjust for energy intake.

**Table 3:** Correlations between the 1-day DDS and nutrient adequacy ratios among the participants (*n* = 330)

	Pre-harvest season (*n* = 127)		Post-harvest season (*n* = 203)	
Nutrient adequacy ratio	Adjusted Pearson’s correlation coefficient^a^	*p*	Adjusted Pearson’s correlation coefficient^a^	*p*
Calcium	0.24	<0.01	−0.11	0.23
Iron	0.09	0.25	−0.09	0.32
Zinc	−0.01	0.91	−0.03	0.76
Vitamin C	0.37	<0.01	0.13	0.15
Thiamin	0.02	0.79	0.03	0.77
Riboflavin	−0.04	0.65	0.13	0.18
Niacin	0.05	0.51	−0.11	0.23
Folate	0.15	0.06	0.17	0.07
Vitamin A	0.46	<0.01	0.13	0.17
Vitamin B_12_	−0.06	0.40	−0.19	0.04
Vitamin B_6_	0.18	0.02	0.01	0.90
Mean adequacy ratio	0.22	<0.01	−0.04	0.65

a All data were log-transformed to meet the criteria for Pearson’s correlation. Partial correlations were conducted to adjust for energy intake.

## Discussion

To our knowledge this is the first study to assess the seasonality of MDD-W and correlations between DDS and nutrient adequacy among pregnant females as a separate group.

Several findings suggest that dietary intakes are sensitive to seasonality in this low-resource setting. The proportion of participants who achieved MDD-W was 24 percentage points higher in the post-harvest season, when food availability is generally sufficient for most households.

In addition to a higher proportion of participants achieving MDD-W, the mean DDS was also higher in the post-harvest season. Seasonal changes in dietary diversity have been documented in other developing countries, but findings on the effect of seasonality on dietary diversity are inconsistent (10). While a study among pregnant women in Bangladesh found decreasing dietary diversity during the lesser lean season and in the month immediately after, some studies have found higher food group diversity in the lean season, as a result of increased intake of wild foods and other available foods (20–22). Findings, including ours, indicate that such adaptations resulting in increased dietary diversity do not occur in the Malawian setting (3, 23). It is important to keep in mind that increased diet diversity in this setting might not lead to significant increases in intakes of nutrients or energy if the foods are eaten in very small amounts (10). In this current study, more than 90% of participants were at risk of inadequate intakes of calcium, iron, zinc, riboflavin, folate, and vitamin A in both seasons. Gaps between intakes and requirements for these nutrients have also been reported elsewhere (8). Although a high percentage of participants were at risk of inadequate intakes of several nutrients in both seasons, the proportion of participants at risk of inadequate intakes of zinc, riboflavin, and thiamin was higher during the preharvest season. A study among rural women of reproductive age in Burkina Faso found that the probability of adequate intakes of most nutrients were higher in the post-harvest season (24). Our findings indicate that seasonality affects both dietary diversity and nutrient adequacy of the participants. As data on vitamin B_12_ intakes indicated zero values, we could not estimate the percentage at risk of inadequate intakes. However, adequacy of this nutrient has been reported as low in other resource-poor areas, and considering the low intake of animal source food in the present setting we can assume that there is a high percentage at risk of inadequate intakes (8, 25).

Previous studies have generally confirmed the positive association between diet diversity and nutrient adequacy among women of reproductive age (8). A study from Bangladesh found DDS to be associated with mean probability of nutrient adequacy for pregnant women as a separate group (25). Few studies have examined the relation between DDS and nutrient adequacy across seasons. Seasonal variations in the strength of association between DDS and mean probability of nutrient adequacy were observed among Zambian children, and in energy adjusted models the associations only remained significant in the early lean season and late post-harvest season, but not in the late lean season (26). However, they found that DDS and mean probability of adequacy was highest in the early lean season, which differs from our findings. In the current study, DDS was only associated with mean NAR in the preharvest season, and the correlation coefficients were similar to both the 3-day and the 1-day DDS. In the preharvest season, both indicators predicted NAR for vitamin B_6_, vitamin C, and vitamin A, with similar correlation coefficients. As the magnitudes of the correlations were similar, there seems to be no advantage to using a 3-day DDS compared to a 1-day DDS as a proxy for nutrient adequacy in this setting. In the post-harvest season, a 3-day DDS was positively correlated with some individual NARs, whereas these correlations were not significant for a 1-day DDS. Surprisingly, the only consistency between these two DDS indicators in the post-harvest season was a negative correlation with NAR for vitamin B_12_. Neither of the two DDS indicators predicted MAR in the post-harvest season.

The correlation coefficients found in the preharvest season are similar to what Arimond et al. found in different sites in Africa (8). Arimond et al. also investigated the performance, usefulness, and limitation of DDS indicators and found that they were suitable for assessment on a population level to highlight problems, motivate interventions, and also for tracking progress, despite a low to moderate correlation with probability of nutrient adequacy (8). However, findings from the current study show that correlations between DDS and the MAR were sensitive to seasonality and in fact were only significant for the preharvest season. In the MDD-W guide to measurement, researchers are encouraged to consider seasonality when performing and interpreting results from food group diversity surveys and to avoid direct comparison if surveys are conducted during different seasons (10). Our findings support the need to consider seasonality in this setting, both because of seasonal changes in DDS and risk of inadequate intakes. Moreover, our results show that DDS was not an appropriate tool to predict nutrient adequacy in the post-harvest season. We thus recommend that DDS be used with care in such settings. More studies are needed to investigate the association between DDS and nutrient adequacy across seasons. If additional studies support our findings, perhaps one should consider a different tool as a proxy for nutrient adequacy among rural pregnant women.

DDS have previously shown a stronger correlation with nutrient adequacy when only including foods consumed at more than 15 g (8). In the present study we did not set a weight limit for foods to be included, which might have led to a weaker correlation. Perhaps we would have detected correlations in the post-harvest season if only foods consumed at more than 15 g were included. Another limitation was that dietary intakes were only measured over a short time period during pregnancy.

The strength of the present study is that we conducted three repeated, non-consecutive quantified 24-h diet recalls for calculating nutrient intakes. In addition, we used multiple reference days for compiling the DDS. Because the participants were visited at three separate times, they only had to recall the previous 24 h, so the challenge of recall bias was minimized compared to methods where for example they are asked to remember the previous 3 or 7 days.

In conclusion, we found positive correlations between DDS and overall nutrient adequacy in the preharvest season, with the use of both a 1-day and a 3-day DDS. For simplicity, we would recommend using a 1-day DDS. However, we did not find a correlation with DDS and overall nutrient adequacy in the post-harvest season. This shows that care should be taken when using DDS as a proxy for nutrient adequacy across seasons in this setting, and the sensitivity of DDS to seasonality must be considered.

## Supplementary Material

Seasonality in associations between dietary diversity scores and nutrient adequacy ratios among pregnant women in rural Malawi – a cross-sectional study
