# Supplementary material for: Seasonality in associations between dietary diversity scores and nutrient adequacy ratios among pregnant women in rural Malawi – a cross-sectional study
Source: Food Nutr Res. 2019 Feb 27;63:10.29219/fnr.v63.2712. doi: 10.29219/fnr.v63.2712 (PMC6397333; doi:10.29219/fnr.v63.2712)
Supplement: Seasonality in associations between dietary diversity scores and nutrient adequacy ratios among pregnant women in rural Malawi – a cross-sectional study [file FNR-63-2712-s001.docx]

**Supplemental figure 1.** Pictorial charts of consumed foods and beverages. The participants were provided with picture charts

prior to dietary recalls and asked to mark all foods and beverages prospectively.****
